# A “No-Code” App Design Platform for Mobile Health Research: Development and Usability Study

**DOI:** 10.2196/38737

**Published:** 2022-08-18

**Authors:** Sam Liu, Henry La, Amanda Willms, Ryan E Rhodes

**Affiliations:** 1 School of Exercise Science Physical and Health Education University of Victoria Victoria, BC Canada

**Keywords:** app development, behavior change technique, health promotion, mobile health, mobile application, application development, design platform, platform development, no-code mHealth app, no-code app, no-code, end user, participatory research, Pathverse, agile, hybrid-agile, software design, software development, software developer, computer science, BCT, behavior change, research tool, research instrument, digital platform, mHealth, mobile app

## Abstract

**Background:**

A challenge facing researchers conducting mobile health (mHealth) research is the amount of resources required to develop mobile apps. This can be a barrier to generating relevant knowledge in a timely manner. The recent rise of “no-code” software development platforms may overcome this challenge and enable researchers to decrease the cost and time required to develop mHealth research apps.

**Objective:**

We aimed to describe the development process and the lessons learned to build Pathverse, a no-code mHealth app design platform.

**Methods:**

The study took place between November 2019 and December 2021. We used a participatory research framework to develop the mHealth app design platform. In phase 1, we worked with researchers to gather key platform feature requirements and conducted an exploratory literature search to determine needs related to this platform. In phase 2, we used an agile software framework (Scrum) to develop the platform. Each development sprint cycle was 4 weeks in length. We created a minimum viable product at the end of 7 sprint cycles. In phase 3, we used a convenience sample of adults (n=5) to gather user feedback through usability and acceptability testing. In phase 4, we further developed the platform based on user feedback, following the V-model software development process.

**Results:**

Our team consulted end users (ie, researchers) and utilized behavior change technique taxonomy and behavior change models (ie, the multi-process action control framework) to guide the development of features. The first version of the Pathverse platform included features that allowed researchers to (1) design customized multimedia app content (eg, interactive lessons), (2) set content delivery logic (eg, only show new lessons when completing the previous lesson), (3) implement customized participant surveys, (4) provide self-monitoring tools, (5) set personalized goals, and (6) customize app notifications. Usability and acceptability testing revealed that researchers found the platform easy to navigate and that the features were intuitive to use. Potential improvements include the ability to deliver adaptive interventions and add features such as community group chat.

**Conclusions:**

To our knowledge, Pathverse is the first no-code mHealth app design platform for developing mHealth interventions for behavior. We successfully used behavior change models and the behavior change technique taxonomy to inform the feature requirements of Pathverse. Overall, the use of a participatory framework, combined with the agile and hybrid-agile software development process, enabled our team to successfully develop the Pathverse platform.

## Introduction

Advancements in internet-enabled digital devices (eg, smartphones and wearables) and improved access to these devices have led to the rapid growth of mobile health (mHealth) research [1]. Due to the flexibility and scalability of mHealth technology, there has been tremendous interest among researchers and public health agencies in leveraging mHealth for promoting a healthy lifestyle and preventing chronic diseases [1-4]. Previous studies have shown that mHealth interventions can be efficacious in improving physical activity and healthy eating behaviors and reducing sedentary behavior [1,2,5]. However, research on the optimal ways to design these mHealth interventions to maximize effectiveness for different health conditions and population groups is still in its infancy. A recent meta-analysis found that efficacious mHealth apps that aimed to improve diet and physical activity and reduce sedentary behavior used a variety of behavior change theories and behavior change techniques (BCTs). BCTs are strategies that help individuals change their behavior; thus, these strategies are critical to creating effective and replicable behavior interventions [6]. Some efficacious apps incorporated BCTs such as motivational messages, rewards, gamification in the form of exergames, social support through interaction with peers, and friendly team challenges [7]. Meanwhile, other effective interventions have shown that using tailored health advice, goal setting, self-monitoring, and performance feedback in an app’s design can lead to greater intervention effects [7-10]. Overall, more research is needed to realize the full potential of mHealth technology.

One of the challenges facing researchers conducting mHealth intervention research is the cost and time required to develop and maintain mobile apps [11]. The cost to develop these customized mobile apps (eg, behavior health counselling interventions, daily diary survey studies, and self-monitoring apps) can range broadly. Even an app with few features can cost between US $70,000 and $100,000 and take 3 to 6 months to develop [12]. Furthermore, the app development cost often does not fit into the budget of government research grants (eg, those provided by the National Institute of Health Research or the Canadian Institute of Health Research). Existing mobile app development kits, such as Apple’s ResearchKit and Android’s ResearchStack, have attempted to improve the app development process for researchers [13,14]. However, these frameworks still require significant software programming to develop apps and often require researchers to hire specialized software developers to develop iOS and Android apps. Due to the rapidly evolving digital technology space, these challenges can be a significant barrier to developing relevant mHealth knowledge in a timely manner [15]. The need for research tools to help generate rapid and relevant research knowledge has been a long-noted issue in health research and a solution is desperately needed [16].

An innovative solution to overcome these mHealth app development challenges facing researchers has recently arisen: “no-code” development platforms [17]. A no-code mHealth research app development platform could enable researchers with no previous software programming skills to create apps through a graphical user interface (UI). Similarly to using no-code tools such as Squarespace to create websites [18], researchers could use a no-code mHealth design platform to create multiple versions of an app to evaluate their effectiveness in various conditions. Researchers could use drag and drop tools to select the required BCTs (eg, self-monitoring and goal setting tools) needed for a behavior change framework. We believe a no-code app design platform could significantly expedite the mHealth app development process and reduce the time and cost required. Currently, there is a lack of a no-code app design platform explicitly designed for researchers to develop mHealth behavior interventions. Thus, the purpose of this paper is to describe the development process and the lessons learned in building a no-code mHealth app design platform, Pathverse.

## Methods

We used a participatory research framework to develop a no-code mHealth app design platform called Pathverse [19]. A participatory framework is a method that involves active collaboration between end users (ie, researchers) and software developers at various stages of development to ensure that the final product is relevant and useful [20,21]. This study was divided into four phases: (1) determination of features required for a no-code mHealth design platform; (2) development of the platform; (3) gathering of user feedback; and (4) implementation of user feedback to further refine the platform. The entire development process took place between November 2019 and December 2021. A summary of the development timeline and activities is shown in Table 1.

**Table 1:** Development phases of the web-based program.

Phases	Activities	Dates
(1) Determine feature requirements	Determine features required for the “no-code” mHealth design platform, Pathverse	Nov 2019-Mar 2020
(2) Develop the platform	Use the Scrum development framework to design the Pathverse platform	May 2020-Dec 2020
(3) Gather user feedback	Usability and acceptability testing	Feb 2021-May 2021
(4) Implement user feedback	Revise the Pathverse platform based on usability and acceptability testing	Sep 2021-Dec 2021

### Phase 1: Determine Feature Requirements (November 2019-March 2020)

mHealth researchers (n=13) with various levels of research experience (eg, students, early career researchers, and senior researchers) and software developers (n=4) were involved in identifying key software features for the no-code app design platform, Pathverse. The team members’ demographics are presented in Multimedia Appendix 1, Table S1. The mHealth research team’s expertise included mHealth app development and evaluation, behavior science, psychology, health promotion, and usability testing. The software development team expertise included Python, JavaScript, Dart, and the Scrum development process. We performed an exploratory web search using Google with the search term *no-code mHealth app builders* to determine whether such mHealth app builders existed. In addition, we performed a literature search to determine mHealth apps features that were associated with intervention effectiveness. Specifically, MEDLINE, PubMed, EMBASE, and PsycINFO were searched for articles published from January 2009 to December 2019 with the following key words: (*mobile health* OR *mHealth* OR *internet intervention* OR *web-based interventions*) AND (*effectiveness* OR *efficacy*) AND (*features* OR *characteristics* OR *behaviour change techniques* OR *theories*) AND (*systematic review* OR *literature review* OR *meta-analysis*). Our interdisciplinary team met regularly throughout this phase to brainstorm features (eg, self-monitoring tools and goal setting) required to build mHealth apps. In order to ensure that these features met the researchers’ requirements for building mHealth apps, our team used the multi-process action control framework (M-PAC) as a theoretical template model and used physical activity change as the template outcome behavior. The M-PAC framework has been shown to be effective in promoting physical activity [22-24]. M-PAC emphasizes a social cognition approach to intention formation, the adoption of action control through self-regulation, and an action control maintenance phase once a behavior becomes habitual and self-identified [23]. One advantage of the M-PAC model is its ability to address the “intention to behavior” gap, which poses a particular challenge for individuals adopting a new lifestyle, because almost all individuals joining mHealth app interventions have already formed an intention to adopt a healthy lifestyle [23,25,26]. Finally, we matched the proposed BCTs to the M-PAC mechanisms of action [22], which guided the features for the Pathverse app development platform. A list of key features and a mock-up design of the Pathverse platform were developed by the end of this phase.

### Phase 2: Develop the Platform (May-December 2020)

We used the Scrum framework to develop the Pathverse platform [27]. This agile software framework uses an iterative approach that allows for valuable input from end users throughout the software development cycle. Scrum uses predefined short-sprint cycles that usually last from 2 to 4 weeks. Each sprint cycle consists of design, implementation, evaluation, and planning for the next sprint. The Scrum framework enables the development team to create the first version of the software at the earliest stage of the development process. Furthermore, regular meetings throughout the development cycles enable end users to provide valuable feedback and make rapid adjustments throughout the development cycles.

We used a 4-week sprint cycle in this project. We aimed to produce a working version of the platform in about 7 months (ie, 7 sprint cycles). The key members in the Scrum team were an mHealth researcher (the product owner, SL), the Scrum master (HL), and the software development team. End users with various levels of mHealth research experience (researchers, research assistants, and students) were involved during each sprint. The Scrum team presented the completed platform features and discussed goals for the next sprint with the team at the end of each sprint.

### Phase 3: Gather User Feedback (February-May 2021)

Similarly to our previous studies [28,29], we gathered user feedback by assessing the usability and acceptability of the Pathverse platform. Usability and acceptability assessments are part of a technique in user-centered interaction design to evaluate how researchers interact with the platform; we used this approach to evaluate whether Pathverse met its intended requirements. We used a convenience sample of health researchers (n=5) who were interested in using or had used mHealth applications in their research. Participants were required to have not previously used the Pathverse platform. Due to the COVID-19 pandemic, the assessments were conducted using video calls. A week prior to the video call, participants were given access to the platform and were asked to use it to build a mobile app program aimed at promoting a healthy lifestyle. During the video call, we conducted a structured interview to gather feedback on what the user liked and disliked about the platform and to determine areas needing improvement. The qualitative interview data were summarized using thematic analysis to identify areas for improvement. Participants were also asked to complete a questionnaire evaluating the likeability and usefulness of the platform. The questionnaires were adapted from an mHealth app usability questionnaire that assesses the likability and usefulness of the platform [30]. The score had a scale ranging from 0 to 10, with 10 indicating “strongly agree,” and 0 indicating “strongly disagree.”

### Phase 4: Implement User Feedback (September-December 2021)

Based on user feedback, our team planned for an additional phase of development. During this phase, we used the V-model software development process. This method combines traditional sequential development methodology (eg, the waterfall method) with feedback mechanisms in the agile development process (eg, Scrum) to ensure that the new features added work appropriately [31]. The V-model software development process was chosen instead of the Scrum method due to the well-defined project requirements and the smaller project size [31]. Our team used the following development stages: (1) requirement analysis (ie, gathering project requirements from researchers), (2) system and architectural design (ie, determining the critical software components required for the final product), (3) module design (ie, determining the critical modules for the software components identified), and (4) coding (ie, starting to program the modules). We also conducted validation testing for each development stage to ensure that the platform worked appropriately. The validation testing consisted of the following: (1) unit testing (performed by the software team to eliminate system bugs during the coding phase), (2) integration testing (performed by the software team to ensure the new features developed worked appropriately with the existing platform), (3) system testing (conducted by the researchers to ensure the development met the build requirement), (4) user acceptance testing (performed by the research team to ensure the platform was ready for use in the real world).

### Ethical Considerations

Informed consent was obtained from participants completing the usability and acceptability testing. This study was approved (17361) by the Human Research Ethics Board at the University of Victoria.

## Results

### Phase 1: Determining Feature Requirements

Our multidisciplinary team of researchers and software developers met regularly to determine requirements and features for the Pathverse platform. A summary of the activities conducted at each meeting is shown in Table 2. Our exploratory Google web search revealed that there was a lack of no-code mHealth app development tools designed for researchers. A literature review suggested that an mHealth app platform would need a variety of software features in order to deliver a wide range of BCTs [3,7,32-34]. For example, a review of BCTs in 40 exercise and dietary apps showed that the apps included an average of 8.1 (range 2-18) techniques [35]. Commonly included BCTs were “provide instruction” (33/40 of apps, 83%), “set graded tasks” (28/40, 70%), “prompt self-monitoring” (24/40, 60%), and “model/demonstrate the behavior” (24/40, 53%). At least one of the following 3 BCTs was also included in 55% (22/40) of the apps: “provide opportunities for social comparison,” “plan social support/social change,” and “prompt identification as a role model” [35]. A more recent systematic review suggested that prompts and cues, personalization, goal setting, and action planning were the most common BCTs used in effective mHealth trials to improve lifestyle behaviors and chronic condition management [36]. However, the optimal number and combinations of BCTs needed for effective mHealth interventions would most likely depend on the underlying theoretical approach and the proposed mechanisms of action. Thus, this reinforces the need for the Pathverse platform to provide researchers with the flexibility of building mHealth apps with various BCTs for a chosen theoretical framework (eg, M-PAC, self-determination theory, or theory of planned behavior).

Our team generated a list of potential Pathverse features by building a mock-up app using the M-PAC framework as a template theoretical framework and physical activity as the behavior change outcome. Similarly to our previous study, our team then matched the BCTs required to implement the physical activity app using the M-PAC framework [28].

The final platform features included the ability to (1) design customized and interactive multimedia content in the app; (2) set flexible content delivery logic (eg, delay the time release or only show new lessons when completing the previous lesson); (3) deploy customized surveys to the participants; (4) provide personalized self-monitoring trackers (ie, daily steps); (5) enable participants to set goals; (6) implement customized app notifications to remind participants of any new mHealth intervention content; (7) provide gamified points and badges; and (8) enable participants to share progress made on their social media accounts (eg, Instagram and Facebook). Table 3 shows how these Pathverse features could potentially be used to deliver the BCTs listed in the Coventry, Aberdeen & London—Refined (CALO-RE) taxonomy [37].

**Table 2:** A summary of the activities conducted during the meetings.

Date	Activities
November 8, 2019	Discussed common features used in current popular mHealth lifestyle promotion apps
December 12, 2019	Brainstormed potential mHealth content and features required for the platform based on an example physical activity promotion app that used the multi-process action control framework Received feedback from researchers on app mock-ups and discussed the user journey for researchers to create apps Provided feedback on potential app logic needed to deliver multimedia content in an app
January 10, 2020	Compiled a wish list of features for an mHealth app builder platform, which included multimedia content delivery, messaging, online community, self-monitoring tools, wearable integration, adaptive intervention delivery logic, gamification features (eg, awards, points, and competitions), diaries, virtual lockers to store memories of accomplishments, surveys, reminders and notifications, goal setting, team challenges, quizzes, the ability to customize the app UI^a^ (eg, color, fonts, and layout), a means of tracking app usage, and a mechanism for online consent
January 30, 2020	Created several UI designs of a “no-code” app design platform Received design feedback from end users
February 14, 2020	Further refined UI designs and discussed the user journey and potential ways researchers could interact with the no-code app design platform to create mHealth apps Discussed potential privacy and security measures that the platform needed to consider Brainstormed and finalized the name of the no-code app design platform: Pathverse
March 12, 2020	Finalized a list of features that our team would attempt to include for the first version of the no-code app design platform Estimated software development timeline and the number of software developers required

^a^User interface.

**Table 3:** Behavior change techniques that can be implemented using the identified Pathverse features.

Pathverse features	Potential behavior change techniques that could be implemented using the proposed Pathverse features. The numbers in parentheses refer to behavior change techniques in the Coventry, Aberdeen & London—Refined taxonomy [37].
(1) Ability to design customized multimedia content (eg, text, pictures, video, and interactive quizzes) on various app pages; the content can be organized into “lessons” depending on the intervention curriculum (for example, lesson 1 might discuss the benefits of physical activity and lesson 2 might provide information on setting graded goals)	Provide information on consequences of behaviour in general (1) Provide information on the consequences of behaviour to the individual (2) Provide information about others’ approval (3) Provide normative information about others’ behaviour (4) Barrier identification/problem solving (8) Set graded tasks (9) Prompt review of behavioural goals (10) Prompt review of outcome goals (11) Prompt rewards contingent on effort or progress towards behaviour (12) Shaping (14) Prompting focus on past success (18) Prompting generalization of a target behaviour (15) Prompt self-monitoring of behavioural outcome (16) Provide information on where and when to perform the behaviour (20) Provide instruction on how to perform the behaviour (21) Model/demonstrate the behaviour (22) Teach to use prompts/cues (23) Environmental restructuring (24) Fear arousal (32) Prompt self talk (33) Prompt use of imagery (34) Relapse prevention/coping planning (35) Stress management/emotional control training (36) Motivational interviewing (37) Time management (38) General communication skills training (39) Prompt identification as role model/position advocate (30) Facilitate social comparison (28)
(2) Set program delivery logic for the content created (for example, a new program lesson can be delivered every week)	Provide feedback on performance (19) Use of follow-up prompts (27)
(3) Deploy customized surveys to the participants; the surveys can include multiple choice answers, Likert scales, and drop-down or open-ended questions	Barrier identification/problem solving (8) Prompt self-monitoring of behavioural outcome (16) Facilitate social comparison (28)
(4) Track physical activity–related outcomes from participants’ fitness trackers; data will be automatically synchronized from trackers connected to Apple or Google Health	Prompt self-monitoring of behaviour (16)
(5) Enable participants to set personal goals; participants can also receive reminders about the goal due date	Goal setting (behaviour) (5) Goal setting (outcome) (6) Action planning (7) Set graded tasks (9) Prompt review of behavioural goals (10) Prompt review of outcome goals (11)
(6) Implement customized app notifications to remind participants of any new mHealth intervention content	Prompt review of behavioural goals (10) Prompt review of outcome goals (11) Prompt practice (26)
(7) Provide gamified points and badges^a^	Prompt rewards contingent on effort or progress toward behaviour (12) Provide rewards contingent on successful behaviour (13) Shaping (14) Stimulate anticipation of future rewards (40)
(8) Enable participants to share progress made on their social media accounts (eg, Instagram and Facebook)^a^	Provide information about others’ approval (3) Facilitate social comparison (28) Plan social support/social change (29) Prompt identification as role model/position advocate (30)

^a^These features were not developed in the Pathverse app (version 1.5).

### Phase 2: Platform Development

The Scrum team met with researchers throughout the sprint cycles to gather user feedback and plan the tasks to be completed by the end of the next phase. A summary of the activities completed in each Scrum phase is described below. The commit history of the software development process can be found in Multimedia Appendix 2.

#### Sprint 1

**Figure 1:**
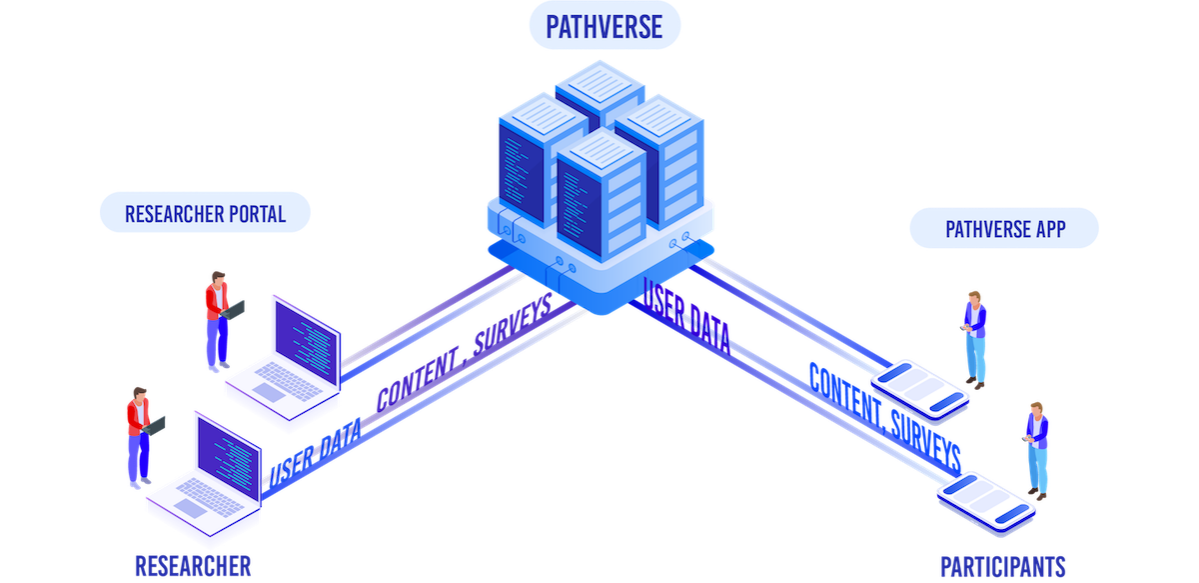
Pathverse platform architecture.

The first sprint started with determining the Pathverse platform architecture required to implement the key features identified in phase 1. The platform consisted of 3 main components: the Pathverse researcher web portal, the Pathverse participant app (available in both the iOS and Android app stores), and the backend application program interface (API) server and databases (Figure 1). The researcher portal enabled a researcher to create mHealth apps. The research participants could then download the Pathverse app to access the intervention. The API server acted as an intermediary between the database and the frontend interfaces by relaying information back and forth between storage and users. In this stage of the sprint, our team planned and designed the foundations of the 3 components. This started with determining all the types of data that were going to be used within these components. With the data structure decided, our team worked on user flow and UI for each of the components. Finally, our team finalized platform security. At the end of this phase, researchers and our programming team met to finalize the platform architecture to start development.

#### Sprint 2

The programming team simultaneously coded the components of the Pathverse platform. At the end of this sprint, the programing team developed a prototype version of the dashboard of the Pathverse researcher web portal using React.js, a JavaScript library licensed from the Massachusetts Institute of Technology [38]. The login and home screen of the participant app used Flutter [39], and the Pathverse API server used Django, an open-source Python web framework (Django Software Foundation). Along with the visuals, the team also completed designing the platform’s database, optimized relationships within the database, and added data serializers and authentication functions. Researchers and the programming team met at the end of the phase to review the preliminary UI designs of the web portal and the participant app.

#### Sprint 3

The main priorities for this sprint were to finish developing the feature that enabled researchers to upload customized multimedia content for an mHealth intervention and set the delivery logic for the intervention. This was the first time the integrated platform was tested collectively. After sharing a working prototype at the end of this phase, researchers tested the prototype and provided feedback on system bugs and design issues, and they also suggested other features that would improve their experience. The top-priority suggestions included the need to optimize multimedia content (eg, font size and color) for various screen sizes, organize the order of the intervention content delivery using drag and drop, and provide real-time visualization of the multimedia content added to the Pathverse participant app in the research portal.

#### Sprint 4

The programming team attempted to implement the suggestions made by the end users from the previous sprint. Additionally, the programing team completed the customized survey feature. This feature enabled the researchers to collect various types of survey responses (eg, multiple choice, ratings or Likert scales, drop-down questions, and open-ended questions). These features were tested, and various system bugs, UI design issues, and additional features were discussed. Specific main feature modification requests included providing options to enable participants to complete the survey multiple times and randomize the order of the questions. Due to the large quantity of feedback received (for UI and feature requests) and the slower than expected feedback implementation, our team decided not to develop 2 features: gamified points and social sharing. This was to ensure that a working prototype of the Pathverse platform could be delivered on time.

#### Sprint 5

The programming team developed and implemented the self-monitoring tool for step tracking in the participant app. This allowed the Pathverse app to connect to Apple or Google Health wearable devices and display a user’s daily step data. The end users worked with the development team to provide feedback on how the wearable data were displayed in the app. The end users provided the feedback that participants should also be able to display other health metrics, including blood pressure, weight, and daily active minutes.

#### Sprint 6

The programming team completed the development of goal setting and customized app notification features during this sprint. The goal-setting features enabled the participants to set customized personal goals and customized reminders for goal due dates. The customized app notification enabled researchers to set personalized app reminders whenever new app content became available to the participants. At the end of this phase, the programing team presented the first beta version of the platform to the end users. Due to time constraints, the programming team could not implement the feature that allowed the participant app to display all the health metrics requested, such as blood pressure and weight. A prototype of the daily active minutes feature was added. Our team decided that the next sprint would focus on conducting quality assurance (QA) testing.

#### Sprint 7

**Figure 2:**
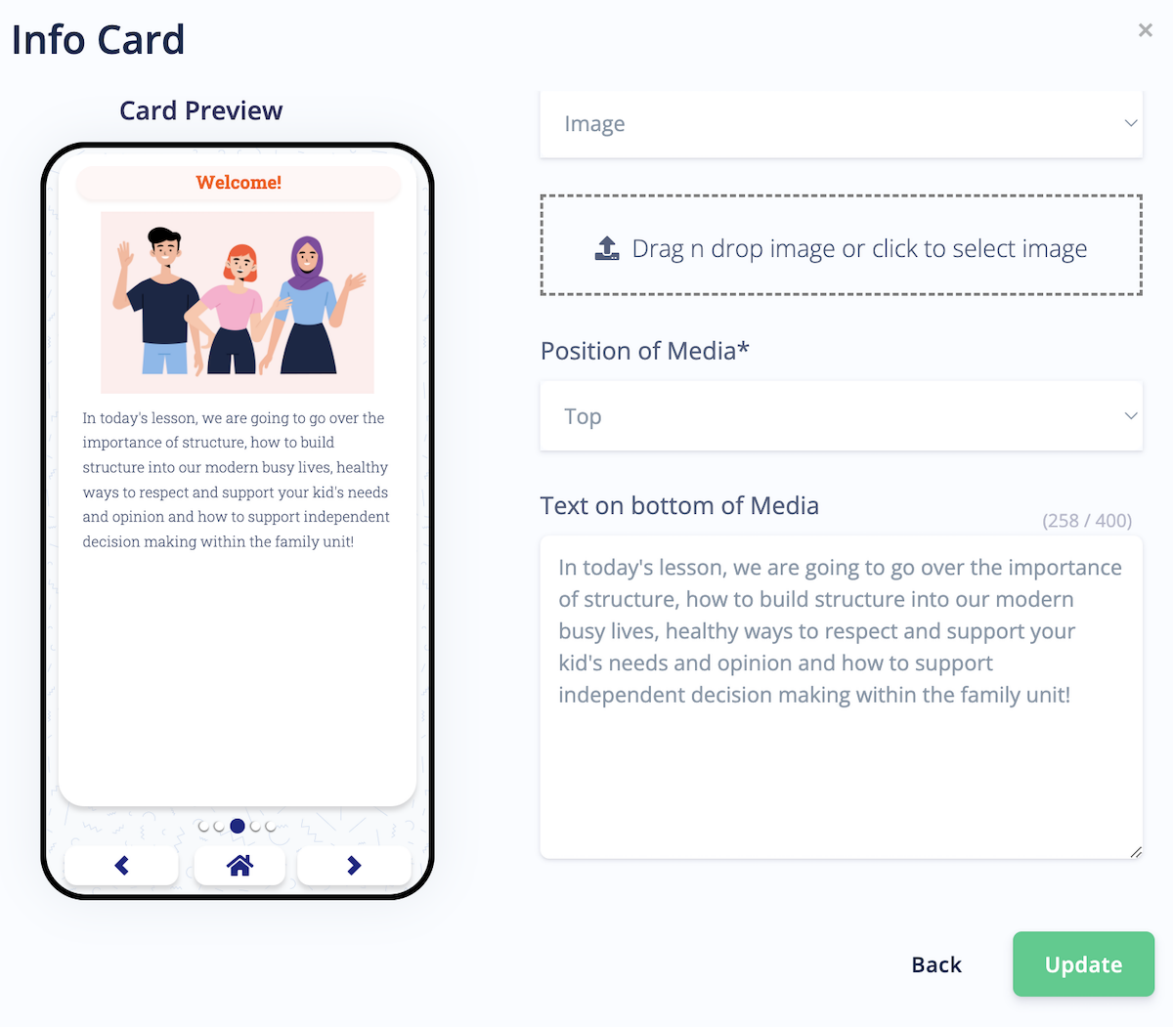
Pathverse researcher portal for creating mHealth app content.

**Figure 3:**
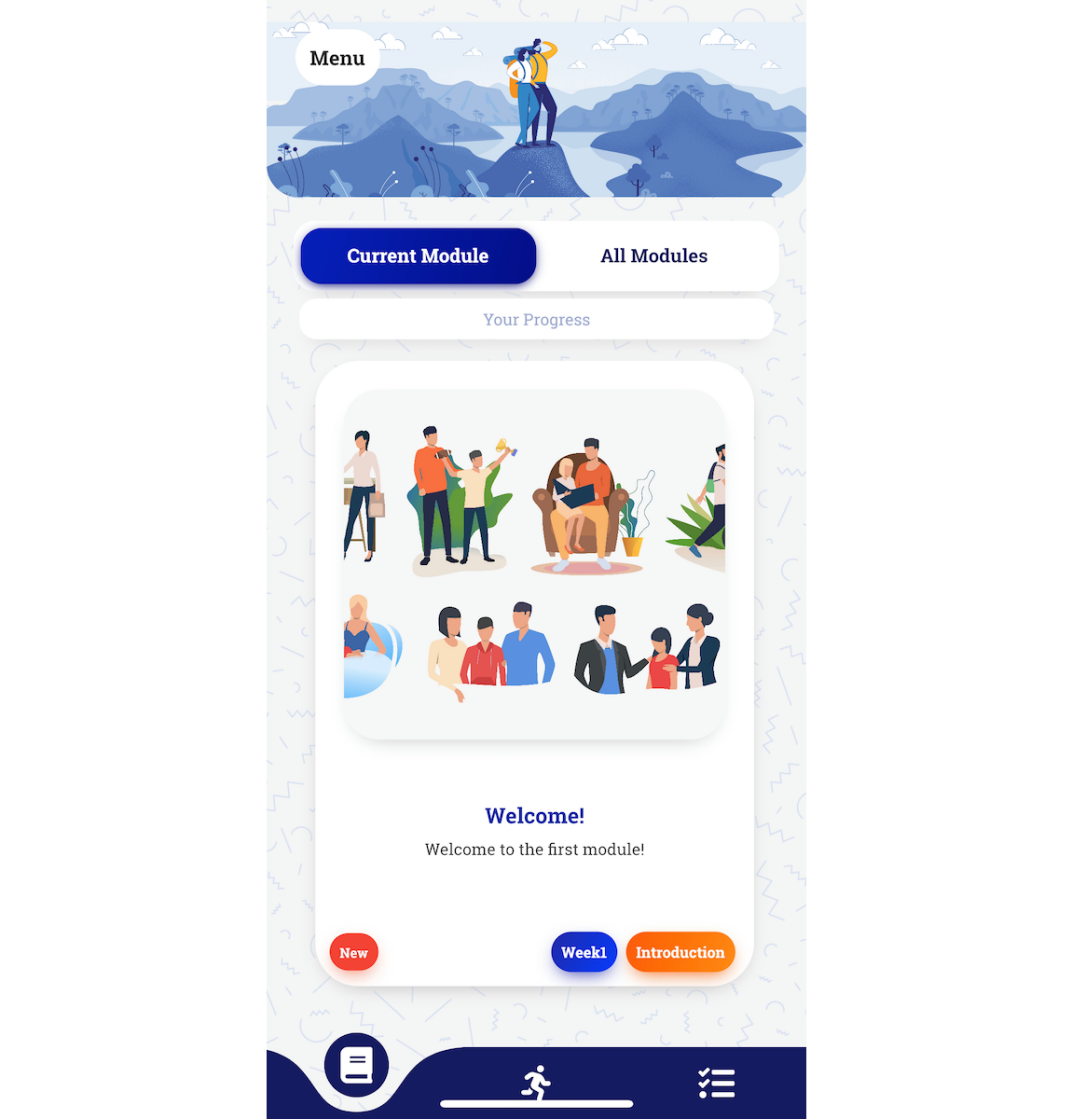
Screenshot of the Pathverse participant app.

The primary goal of this sprint was to conduct QA testing prior to launching the Pathverse platform and submitting the app to the Apple App Store and Google Play Store. The end users and programming team generated a list of system bugs while testing the various features that were developed (eg, multimedia tools, survey tools, and self-monitoring tools). The programming team and the end users met weekly during this sprint to discuss solutions to resolve known system bugs. The app (version 1.0) was officially submitted to the iOS and Android app stores at the end of this sprint. Figure 2 shows screenshots of the Pathverse research portal for creating mHealth app interventions and Figure 3 shows the Pathverse participant app.

### Phase 3: Gathering User Feedback

We invited 5 participants to provide feedback on the Pathverse platform. The demographic characteristics of the researchers are shown in Multimedia Appendix 1, Table S2. Overall, all participants had previous experience with mHealth research; 3 of 5 were from a different research institution from the platform development team. Overall, the platform received high scores for likeability (mean score 8.2, SD 2.2, range 4-10) and usefulness (mean score 8.3, SD 1.5, range 6-10). See Multimedia Appendix 1, Table S3 for a descriptive summary (with the mean, SD, range) of the questionnaire items used to evaluate platform likeability and usefulness.

The most helpful features identified by the users included easy navigation for both the research portal and participant app and the ability to download app usage and survey data. Potential improvements included the ability to deliver multiple surveys throughout the day, add the ability to deliver adaptive interventions, and add features such as community group chat. A summary of the feedback received is shown in Table 4.

**Table 4:** Summary of feedback received in phase 3.

Questions	Summary of feedback (illustrative quotes)
What did you like about the app?	*The participant’s app layout was easy to navigate.* *The self-monitoring tools for physical activity were useful and it was great that can be integrated with Apple and Google Health.* *The user-interface design is clean and logical.* *It is available on both IOS and Android.*
What did you dislike about the app?	*Slow load time when multiple modules were added.* *Text font size was too small on some pages.* *There is limited character space per page. Some text/title are cut off in the app.* *There are some spacing/formatting issues. Not sure if this can be fixed on the admin portal or a display issue.*
What changes do you think can help improve the app?	*Greater ability to customize app layout, color, font.* *Add community chat features, gamification.* *Current goals can only be marked as complete. The ability to mark current goals as incomplete and need time to revisit can be helpful.* *Zoom/video chat integration.* *More self-monitoring tools can be helpful (e.g. weight training log, diet log).*
What did you like about the research web portal?	*Good research portal navigation. It was easy to use the multimedia content, quizzes, surveys to the mHealth app.* *Easy to use research portal console to enrol research participants* *It was great to see the updates made to the app is reflected in real-time.* *The ability to download app usage and survey data.*
What did you dislike about the research web portal?	*The self-monitoring tools are pretty limited. It will be great to add more monitoring tools and integrate with other wearables.* *Sometimes the web portal will not be able to save the order of the modules. Autosave will be helpful.* *Finding the right image size for the app graphics is challenging.* *Not knowing how long the title or text should be before it gets cut off in the app.* *Can’t change the font size or color.* *Not sure the function of the “tags”. Need better instructions.*
What changes do you think can help improve the research web portal?	*The ability to deliver multiple surveys throughout the day. This can greatly expand the survey feature to be used for a daily diary or ecological momentary assessment study.* *The ability to choose whether to display self-monitoring tools in the participant app. Not all mHealth studies (e.g., daily diary/EMA studies) need to show participants their daily steps.* *The app usage data download can be formatted in a way that is easier for analysis (e.g. long vs wide format).* *The ability to download third party wearable data from the platform.* *Should consider adding adaptive intervention delivery logic.* *Add rich text card will be helpful.* *Change the app preview to look like a phone can enhance the preview experience.*

### Phase 4: Implementing User Feedback

We applied the V-method of software development. The requirement analysis phase occurred in September 2021. Based on the user feedback from the previous phase, our team determined three main requirements that we would implement given the availability of resources: (1) expand survey functionalities so that multiple surveys could be delivered throughout the day, (2) improve the data download format (eg, allow a longer data structure format) for easier analysis and postprocessing, and (3) provide the ability to customize whether to use the self-monitoring features, as not all mHealth studies require this feature. The system and architecture design and the module design phase took place during the last week of September 2021. The programming team determined the main modules to be developed to meet the program requirements. These modules included customized survey release times in the researcher web portal, a display of the various surveys in the participant Pathverse app, the ability to download survey data in .csv format, the ability for researchers to choose whether to collect wearable and survey data in the researcher web portal, and revision of the UI design for the participant app to not display self-monitoring tools. Based on these module designs, the software team initiated the coding phases from October to November 2021. The validation testing to resolve system bugs took place in December 2021. Pathverse (version 1.5) was released to the app stores at the end of this phase. Video demonstrations of the functionality of the platform were made available online [40].

## Discussion

### Principal Findings

This study describes the development process of a no-code mHealth app design platform for researchers. To our knowledge, this platform (Pathverse) is the first no-code mHealth app design platform for developing mHealth behavior interventions. This platform has the potential to enable researchers with no previous software programming skills to design mHealth intervention apps. Consequently, this should help reduce the time and cost required to develop mHealth interventions. Our team used a behavior theory framework (M-PAC) and the BCT taxonomy to inform the design of the various software features in the first version of the Pathverse platform. These features can offer researchers the flexibility to design mHealth interventions with various BCTs, depending on the behavior theory or the mechanisms of action. The participatory development methods used in this project allowed our team to ensure that feedback from end users (researchers) was incorporated throughout the development phases. Despite receiving helpful feedback (eg, the social wall, gamification, and app color and font customization) from the researchers, our team could only address the most important issues given resource availability. However, we plan to address all the feedback received in future development.

### Comparison With Prior Work

Similarly to previous mHealth software development studies [28,29], our team learned several lessons throughout the development process. First, the use of M-PAC and matching the proposed BCTs to the M-PAC mechanisms of action was effective in gathering feature requirements for the Pathverse platform. This process enabled our team to determine various BCT use cases and ways to implement them using the features developed (eg, multimedia content delivery, program logic, and self-monitoring tools). We believe that future platform development could use a similar process and could benefit from the use of other behavior theories as templates. This may help our team discover new use cases for the features developed.

The software development methods (eg, Scrum and the V-model) used in this study were effective in delivering the product on time. However, our team found that the Scrum method easily led to scope creep, resulting in a buildup of backlog tasks and feature cancellation. For example, after completing the customized survey feature, the research team requested additional survey functionality (eg, randomization of survey choices) in sprint 4. Similarly, they requested additional self-monitoring trackers (eg, for weight and blood sugar) in sprint 6. We also spent a significant amount of time optimizing and making changes to the app UI throughout the Scrum cycles. Future development should set a limit on the number of UI changes that can be made after the initial designs have been approved. Scope creep is a known challenge in agile development environments [16]. There are several contributing factors to scope creep, which include unclear communication, project complexity, quality issues, time constraints, over-optimism, and unwillingness to say “no” to the client [41]. Several strategies have been proposed to prevent scope creep in software development [42]. For example, mapping the impact of a change as a percentage of the time, cost, and quality of the product could help an agile project manager decide whether to accept or reject the change. Future development may consider using similar techniques to control scope creep.

Finally, we learned the need to implement QA protocols throughout the software development phases. We did not designate a specific QA analyst role during the rapid sprint cycles. Thus, some QA issues were not discovered until the product was launched. Adopting QA testing early in the development cycle could help avoid users experiencing software bugs following deployment. An advantage of using the V-model was the systematic approach to QA testing throughout the development stages. Thus, future Scrum software development might consider incorporating a dedicated QA analyst as part of the team.

### Strengths and Limitations

The end users identified several useful features (eg, the online community, adaptive intervention features, and gamification features) that could be implemented in the future to further expand the capabilities of the no-code mHealth app builder tool for researchers. A strength of the study was using the participatory framework throughout the development of the Pathverse platform. This process enabled our team to gather valuable insights into ways to improve the platform. A limitation of the study is that the end users who were involved in designing and testing the platform were mHealth researchers; this may limit the generalizability of our findings beyond this population. Furthermore, the end users provided feedback about platform feature development throughout the development phases. Due to our limited sample size during usability testing, it remains unclear how these features would be used in a larger group of users. Future studies are warranted.

### Conclusion

In summary, this study describes the development process of Pathverse, a no-code mHealth app design platform. The process reinforced the importance of involving end users (eg, researchers) and demonstrated the use of agile and hybrid-agile software development methods to develop mHealth research tools. Our participatory research approach enabled our team to clarify the feature requirements of the Pathverse platform. Overall, we believe that our no-code mHealth app design platform will help researchers decrease the resources required to leverage mHealth technology.

## Appendix

Multimedia Appendix 1 Supplementary Tables S1 S2 S3.

Multimedia Appendix 2 Platform Commit History.

## Abbreviations

API: application program interface
BCT: behavior change technique
M-PAC: multi-process action control
mHealth: mobile health
QA: quality assurance
UI: user interface
