# Supplementary material for: A “No-Code” App Design Platform for Mobile Health Research: Development and Usability Study
Source: JMIR Form Res. 2022 Aug 18;6(8):e38737. doi: 10.2196/38737 (PMC9437789; doi:10.2196/38737)
Supplement: Multimedia Appendix 1 [file formative_v6i8e38737_app1.docx]

Table S1: Demographics Characteristics of the Platform Development Team

| **Variable** | **N=17 (%)** |
| --- | --- |
| Age range |  |
| 20-30 | 13 (76%) |
| 30-40 | 1 (6%) |
| 40-50 | 3 (18%) |
| mHealth Research Team |  |
| Undergraduate Students | 1 (6%) |
| Graduate Students (M.Sc., Ph.D.) | 7 (41%) |
| Post Doc | 1 (6%) |
| Research Coordinators | 2 (12%) |
| Faculty Members | 2 (12%) |
| Software Development team |  |
| >3 years of software development | 1 (6%) |
| <3 years of experience | 3 (18%) |
| Sex (Female) | 6 (35%) |

Note: Data are presented as *n* (percentage).

Table S2: Participant Demographic Characteristics: Usability and Acceptability Testing

| **Variable** | **N=5 (%)** |
| --- | --- |
| Age range |  |
| 20-30 | 1 (76%) |
| 30-40 | 2 (6%) |
| 40-50 | 1 (18%) |
| Current Position |  |
| Graduate Students (M.Sc., Ph.D.) | 2 (41%) |
| Faculty Members | 3 (12%) |
| Sex (Female) | 3 (35%) |

Note: Data are presented as *n* (percentage).

Table S3: Platform Likability and Usefulness Score

| **Likability** | **n=5**  **Mean (SD)** | **Range** |
| --- | --- | --- |
| I like the user-interface of the research admin portal | 8.2 (2.2) | 5-10 |
| I like the user-interface of the participant App | 8.8 (1.8) | 6-10 |
| The research admin portal was easy to use. | 8.0 (2.0) | 5-10 |
| The participant app was easy to use. | 8.6(1.7) | 6-10 |
| The user-interface of the research admin portal allowed me to use all the functions offered by the platform. | 8.4(2.1) | 4-10 |
| Whenever I made a mistake in the research admin portal, I could recover easily and quickly. | 8.2(2.2) | 4-10 |
| Overall | 8.2(2.2) | 4-10 |
| **Usefulness** |  |  |
| The platform would be useful for me as a researcher | 8.6(1.7) | 6-10 |
| This platform has all the functions and capabilities I expected it to have. | 8.4 (1.5) | 6-10 |
| This platform provided an acceptable way to create mHealth research interventions | 8.0 (1.4) | 6-10 |
| Overall | 8.3(1.5) | 6-10 |
